# Organization, Phylogenetic Marker Exploitation, and Gene Evolution in the Plastome of *Thalictrum* (Ranunculaceae)

**DOI:** 10.3389/fpls.2022.897843

**Published:** 2022-05-20

**Authors:** Kun-Li Xiang, Wei Mao, Huan-Wen Peng, Andrey S. Erst, Ying-Xue Yang, Wen-Chuang He, Zhi-Qiang Wu

**Affiliations:** ^1^Shenzhen Branch, Guangdong Laboratory of Lingnan Modern Agriculture, Genome Analysis Laboratory of the Ministry of Agriculture and Rural Affairs, Agricultural Genomics Institute at Shenzhen, Chinese Academy of Agricultural Sciences, Shenzhen, China; ^2^State Key Laboratory of Systematic and Evolutionary Botany, Institute of Botany, Chinese Academy of Sciences, Beijing, China; ^3^College of Ecology and Environment, Hainan University, Haikou, China; ^4^College of Life Sciences, University of Chinese Academy of Sciences, Beijing, China; ^5^Central Siberian Botanical Garden, Russian Academy of Sciences, Novosibirsk, Russia; ^6^Laboratory Herbarium (TK), Tomsk State University, Tomsk, Russia; ^7^Kunpeng Institute of Modern Agriculture at Foshan, Chinese Academy of Agricultural Sciences, Foshan, China

**Keywords:** *Thalictrum*, plastid genome, genome structure, molecular markers, phylogeny

## Abstract

*Thalictrum* is a phylogenetically and economically important genus in the family Ranunculaceae, but is also regarded as one of the most challengingly difficult in plants for resolving the taxonomical and phylogenetical relationships of constituent taxa within this genus. Here, we sequenced the complete plastid genomes of two *Thalictrum* species using Illumina sequencing technology *via de novo* assembly. The two *Thalictrum* plastomes exhibited circular and typical quadripartite structure that was rather conserved in overall structure and the synteny of gene order. By updating the previously reported plastome annotation of other nine *Thalictrum* species, we found that the expansion or contraction of the inverted repeat region affect the boundary of the single-copy regions in *Thalictrum* plastome. We identified eight highly variable noncoding regions—*infA-rps8*, *ccsA-ndhD*, *trnS^UGA^-psbZ*, *trnH^GUG^-psbA*, *rpl16-rps3*, *ndhG-ndhI*, *ndhD-psaC*, and *ndhJ-ndhK*—that can be further used for molecular identification, phylogenetic, and phylogeographic in different species. Selective pressure and codon usage bias of all the plastid coding genes were also analyzed for the 11 species. Phylogenetic relationships showed *Thalictrum* is monophyly and divided into two major clades based on 11 *Thalictrum* plastomes. The availability of these plastomes offers valuable genetic information for accurate identification of species and taxonomy, phylogenetic resolution, and evolutionary studies of *Thalictrum*, and should assist with exploration and utilization of *Thalictrum* plants.

## Introduction

*Thalictrum* L., comprising *ca.* 200 species, is a phylogenetically and economically important genus in the family Ranunculaceae (Tamura, 1995) and is worldwide with main distribution in northern temperate regions. *Thalictrum* plants are rich in benzylisoquinoline-derived alkaloids; at least 250 such compounds have been isolated from 60 species, and most of them show strong biological activities (Zhu and Xiao, 1991). *Thalictrum* plants are used in folk medicine for the treatment of many kinds of diseases by various ethnic groups of China, which has a long history (Wang and Xiao, 1979; Zhu and Xiao, 1989; Wu et al., 1998; Wang et al., 2001). In some place, roots of *Thalictrum* were used as substitutes for *Rhizoma coptidis* to treat enteritis and dysentery (Wu et al., 1998). Furthermore, bearing luxuriant foliage, extended branches, and attractive flowers, *Thalictrum* species have previously been mainly applied as perennial garden plants. At present, the horticultural values of *Thalictrum* plants, such as *Thalictrum delavayi*, *Thalictrum reniforme*, and *Thalictrum grandiflorum* have been widely paid attention with great commercial prospects (Wang and Xiao, 1979).

*Thalictrum* is taxonomically and phylogenetically regarded as one of the most challengingly difficult taxa in plants. Traditionally, *Thalictrum* was classified into 14 sections based on morphological traits such as leaf, flower, and fruit characteristics (Tamura, 1995). Molecular phylogenetic analyses have consistently suggested only that *Thalictrum* is a monophyletic group containing two major clades, based on the nuclear ribosomal internal transcribed spacer (ITS) region (ITS1, ITS2, and 5.8S) and the chloroplast DNA (cpDNA) *rpl16* intron (Soza et al., 2012). Then, a revised phylogeny yielded better resolution based on nuclear ribosomal ITS region, external transcribed spacer (ETS) region, and the cpDNA 3’*trnV-ndhC* (*trnV-ndhC*) intergenic region (Soza et al., 2013). Nonetheless, none of the sections traditionally assigned to the genus (Tamura, 1995) are monophyletic (Soza et al., 2012, 2013). Moreover, numerous species and varieties in *Thalictrum* are poorly defined owing to insufficient field studies and lack of consistent characteristics for diagnostic methods in the literature (Wang et al., 2001). Therefore, further exploiting more stable genetic variations and effective molecular markers in *Thalictrum* species is greatly important for conservation and utilization of the plants from this genus.

The popularity of the ITS region for infrageneric studies within angiosperms is well-known (Baldwin et al., 1995; Hughes et al., 2006; Mort et al., 2007). Levels of ITS sequence divergence within *Thalictrum* are relatively high (Soza et al., 2012, 2013). However, *Thalictrum* exhibits an enormous range of ploidy, from 2n = 2x = 14 to 2n = 24x = 168 (Löve, 1982; Tamura, 1995), with very small chromosomes known as the T-type in Ranunculaceae (Langlet, 1927). In *Thalictrum*, the ITS region is often presented as more than one copy (Soza et al., 2012, 2013). Owing to their haploidy, maternal inheritance, and high conservation in gene content and genome structure, the plastomes have been popular in researches on evolutionary relationships at almost any taxonomic level in plants. Although sequence divergence among the interspecific cpDNAs is generally less than ITS (Hughes et al., 2006; Mort et al., 2007), it is necessary to utilize cpDNA regions that exhibit relatively high rates of substitution in *Thalictrum*. With the advent of high-throughput sequencing technologies, it is now more practical and inexpensive to obtain plastome sequences and to upgrade cp-based phylogenetics to phylogenomics.

In the present study, we sequenced the complete plastid genomes of two *Thalictrum* species by using the next-generation sequencing platform and performed the first comprehensive analysis of *Thalictrum* plastomes by combining these data with previously reported plastomes of other nine species (Park et al., 2015; He et al., 2019, 2021b; Morales-Briones et al., 2019). Our study aims were as follows: (1) to investigate global structural patterns of the 11 *Thalictrum* plastomes; (2) to identify the most variable regions of these plastomes as prospective DNA barcodes for future species identification; (3) to choose more effective molecular markers *via* reconstruction of phylogenetic relationships among the 11 *Thalictrum* species using various makers; and (4) to test for the presence of adaptive evolution in all genes located in the two single-copy regions, and one of the two inverted-repeat (IR) regions by analyses of selective pressure and codon usage bias. The results will provide abundant information for further species identification, phylogenetic, and phylogeographic studies on *Thalictrum*, and will assist in exploration and utilization of *Thalictrum* plants.

## Materials and Methods

### Sample Preparation, Sequencing, Assembly, and Annotation

The sequenced two *Thalictrum* species (*Thalictrum minus* var. *hypoleucum* and *Thalictrum simplex*) are growing in the Beijing Botanical Garden, Beijing, China. Genomic DNA was extracted from fresh leaves and purified using the Tiangen Isolation/Extraction/Purification Kit [Tiangen Biotech (Beijing) Co., Ltd.]. Short insert of 300–500 bp libraries were prepared for sequencing on the Illumina HiSeq X-Ten platform.

Before assembly of the short reads, plastome original reads were extracted by mapping all short reads to the nine plastomes as reference with BWA (Li and Durbin, 2009) and SAMtools (Danecek et al., 2021). Then the two plastomes were *de novo* assembled with SPAdes v3.15.2 (Bankevich et al., 2012) as described in He et al. (2021a). Highly accurate annotation of organelle genomes was performed by using the Organellar Genome GeSeq tool (Tillich et al., 2017) with subsequent manual correction. Three chloroplast genomes from *Thalictrum coreanum* (GenBank accession No. NC_026103), *Thalictrum minus* (NC_041544), and *Thalictrum thalictroides* (NC_039433) were used as reference sequences. The circular plastomes were visualized by using OGDRAW v1.3.1 (Greiner et al., 2019), with subsequent manual editing. We also updated the annotation of plastomes for the other 11 species in this study.

### Detection and Annotation for Plastid Genomic Variations

Multiple sequence alignments of whole plastome sequences from the 11 *Thalictrum* species that have the representatives of the two major clades of this genus in previous studies (Soza et al., 2012, 2013), as well as *Paraquilegia anemonoides* and *Leptopyrum fumarioides* in Thalictreae as outgroups were implemented using MAFFT v7 (Katoh and Toh, 2010) with standard parameters, and further adjusted manually in Geneious v8.0.4 (Kearse et al., 2012). For comparison, the gene order and structure of the 13 plastomes were compared by using IRscope.^1^

To identifying hypervariable regions, the sequence alignment of *Thalictrum* plastomes without outgroups was subjected to a sliding window analysis in DNAsp v6.12.03 (Rozas et al., 2017) to evaluate nucleotide diversity (*π*) of all genes, genes without introns, and intergenic spacer (IGS) regions. Functional annotations for the nucleotide variations were conducted by using snpEff v5.1 (Cingolani, 2012).

### Phylogenetic Analysis

Phylogenetic analyses of *Thalictrum* were performed with maximum likelihood (ML) method in RAxML v8.2.11 (Stamatakis, 2014) with 1,000 replicates under GTRGAMMA model. The analyses were carried out based on the following nine data sets, including the complete plastid DNA sequences, concatenation of 115 IGS regions, concatenation of 114 gene sequences, and six genes and/or their introns and spacers (*rpl16* intron, *ndhC-trnV^UAC^*, *ndhA* intron, *trnL^UAA^-trnF^GAA^*, *rpl32-trnL^UAG^*, and *rbcL*) that have been employed in previous studies on *Thalictrum* (Soza et al., 2012, 2013; Wang et al., 2019).

### Selective Pressure Analysis

Selective pressures were detected throughout the phylogenetic tree of *Thalictrum* for each plastid gene. Nonsynonymous (*d_N_*) and synonymous (*d_S_*) substitution rates of each plastid gene were assessed by using the CODEML program in PAML v4.9 (Yang, 2007). We tested different hypotheses *via* branch models, H0: the one-ratio model (m0), assumes the same *d_N_*/*d_S_* ratio (*ω* ratio) for all branches in the phylogeny, HA: the free-ratio model (m1) that assumes an independent *ω* ratio for each branch. Likelihood ratio tests were used to test each model’s fit. The double log-likelihood difference between the two models (2ΔL) was compared to a chi-square distribution with N–1 degrees of freedom, where N is the number of branches in the phylogeny (Whelan and Goldman, 1999).

### Codon Usage Analysis

The program DNAsp v6.12.03 (Rozas et al., 2017) was used to examine the synonymous codon usage of 79 protein-coding genes in the plastome of *Thalictrum* and to calculate several related parameters such as the effective number codons (ENC), codon bias index (CBI), and relative synonymous codon usage (RSCU). The ENC and CBI are often used to evaluate codon bias at the level of an individual gene (Frank, 1990). RSCU is the observed codon frequency divided by the expected frequency. An RSCU value close to 1.0 indicates that the deviation is not significant (Sharp et al., 1986). Amino acid (AA) frequency was calculated as the percentage of codons encoding the same amino acid divided by the total codons.

## Results

### Genome Features

**Figure 1:**
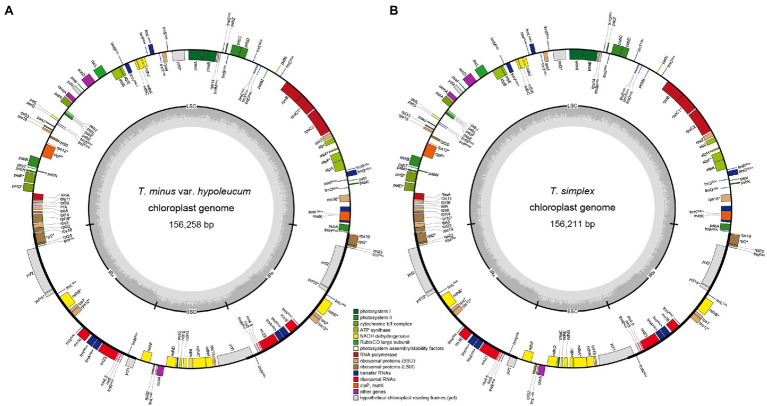
Plastome of *Thalictrum minus* var. *hypoleucum* **(A)** and *Thalictrum simplex* **(B)**. The genes inside and outside of the circle are transcribed in clockwise and counterclockwise directions, respectively. Genes belonging to different functional groups are shown in different colors. The thick lines indicate the extent of the inverted repeats (IR_A_ and IR_B_) that separate the genomes into small single-copy (SSC) and large single-copy (LSC) regions.

**Figure 2:**
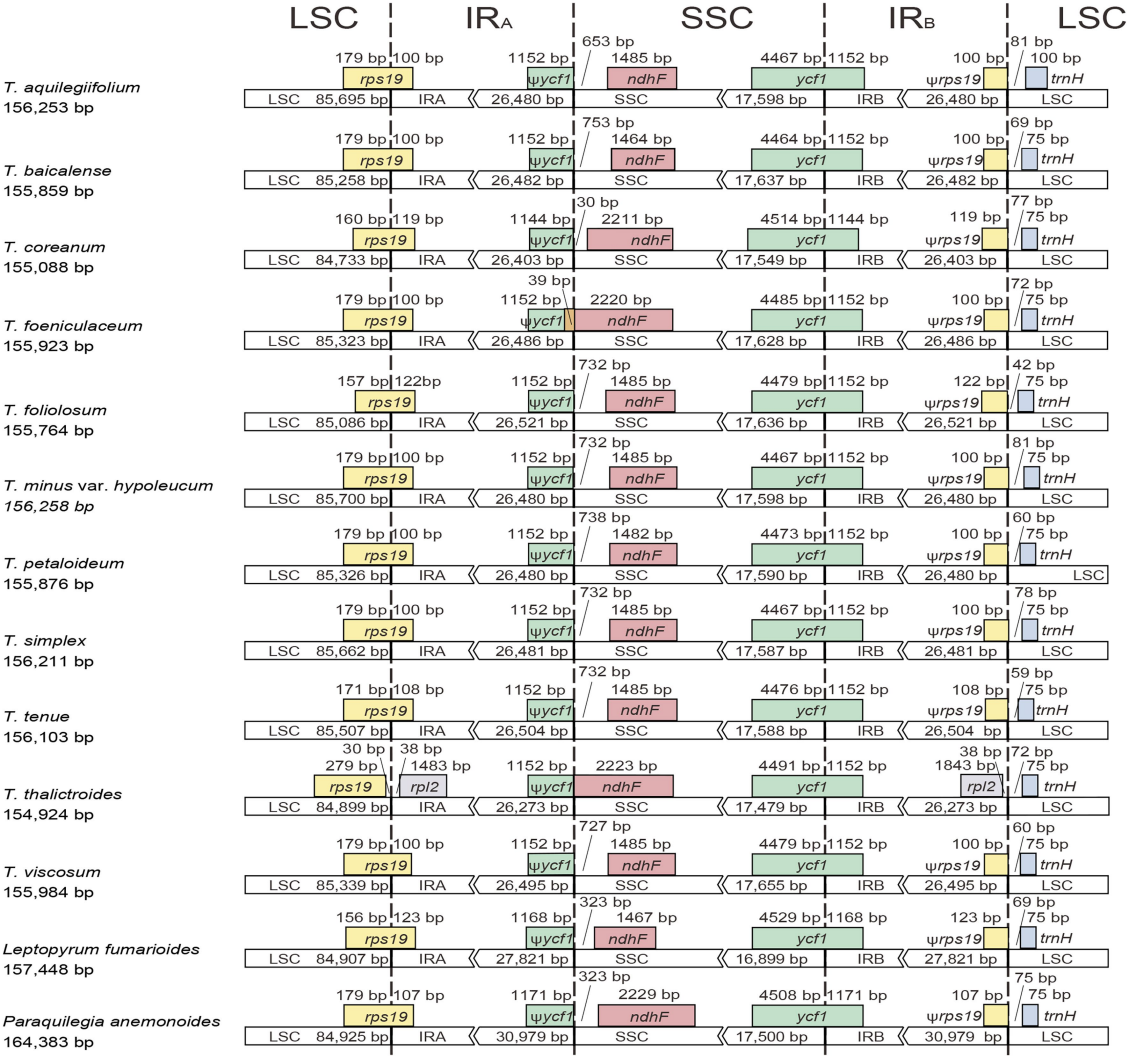
Comparison of LSC, inverted-repeats (IRs), and SSC junction positions among *Thalictrum* plastomes.

The 11 plastomes of the *Thalictrum* species ranged in size from 154,924 bp (*T. thalictroides*) to 156,258 bp (*T. minus* var. *hypoleucum*). All these plastomes displayed the typical quadripartite structure of nearly all land plants, consisting of a pair of inverted repeats (IRs, 26,273–26,521 bp) separated by a single-copy (LSC) region (84,733–85,700 bp) and a small single-copy (SSC) region (17,479–17,655 bp; Table 1). The average GC content was ~38.39%, which is almost identical with each other among the 11 complete *Thalictrum* plastomes. In the IR region, the GC content (43.22%) was found to be much higher than that in the LSC (36.62%) and SSC regions (32.45%). Although overall genomic structure including gene number and gene order were well-conserved (Figure 1), the 11 *Thalictrum* plastomes exhibited obvious differences in the IR-SC boundary regions (Figure 2). The gene *ycf1* spanned the SSC-IR_B_ region while a pseudogene fragment ψ*ycf1* was located at the IR_A_ region with a length range of 1,144–1,152 bp. The gene *rps19* spanned the LSC-IR_A_ region and a pseudogene fragment ψ*rps19* (100–122 bp) was located in the IR_B_ region of all *Thalictrum* species except *T. thalictroides*. At the junction of IR_A_ and SSC regions in most species, the distance between ψ*ycf1* and *ndhF* ranged from 0 to 752 bp, except for that of *Thalictrum foeniculaceum* with an overlap region of 39 bp between ψ*ycf1* and *ndhF*. At the junction of IR_B_ and LSC regions, the distances between ψ*rps19* and *trnH* ranged from 42 to 81 bp.

**Table 1:** Summary of characteristics of plastome sequnences used in the study.

Species	GenBank numbers	Total genome size (GC content)	LSC size (GC content)	IR size (GC content)	SSC size (GC content)	No. total gene (unique gene)	No. protein-coding gene (unique gene)	No. tRNA gene (unique gene)	No. rRNA gene (unique gene)	No. pseudo gene
*Thalictrum aquilegiifolium* L.	MZ442608	156,253 (38.35%)	85,695 (36.55%)	26,480 (43.23%)	17,598 (32.41%)	134 (114)	86 (79)	37 (30)	8 (4)	3
*Thalictrum baicalense* Turcz. ex Ledeb.	MW133265	155,859 (38.39%)	85,258 (36.63%)	26,482 (43.22%)	17,637 (32.41%)	134 (114)	86 (79)	37 (30)	8 (4)	3
*Thalictrum coreanum* H. Lév.	NC_026103	155,088 (38.44%)	84,733 (36.68%)	26,403 (43.25%)	17,549 (32.49%)	134 (114)	86 (79)	37 (30)	8 (4)	3
*Thalictrum foeniculaceum* Bunge	NC_053570	155,923 (38.34%)	85,323 (36.57%)	26,486 (43.21%)	17,628 (32.30%)	134 (114)	86 (79)	37 (30)	8 (4)	3
*Thalictrum foliolosum* DC.	MZ196217	155,764 (38.46%)	85,086 (36.71%)	26,521 (43.22%)	17,636 (32.58%)	134 (114)	86 (79)	37 (30)	8 (4)	3
*Thalictrum minus* var. *hypoleucum* (Siebold & Zucc.) Miq.	OM501079	156,258 (38.35%)	85,700 (36.55%)	26,480 (43.23%)	17,598 (32.41%)	134 (114)	86 (79)	37 (30)	8 (4)	3
*Thalictrum petaloideum* L.	MK253449	155,876 (38.42%)	85,326 (36.64%)	26,480 (43.23%)	17,590 (32.55%)	134 (114)	86 (79)	37 (30)	8 (4)	3
*Thalictrum simplex* L.	OM501080	156,211 (38.36%)	85,662 (36.56%)	26,481 (43.22%)	17,587 (32.46%)	134 (114)	86 (79)	37 (30)	8 (4)	3
*Thalictrum tenue* Franch.	MK253448	156,103 (38.37%)	85,507 (36.59%)	26,504 (43.2%)	17,588 (32.43%)	134 (114)	86 (79)	37 (30)	8 (4)	3
*Thalictrum thalictroides* (L.) A. J. Eames & B. Boivin	NC_039433	154,924 (38.43%)	84,899 (36.66%)	26,273 (43.26%)	17,479 (32.5%)	133 (114)	86 (79)	37 (30)	8 (4)	2
*Thalictrum viscosum* W. T. Wang & S. H. Wang	MZ442609	155,984 (38.38%)	85,339 (36.63%)	26,495 (43.2%)	17,655 (32.36%)	134 (114)	86 (79)	37 (30)	8 (4)	3
*Leptopyrum fumarioides* (L.) Rchb.	NC_041542	157,448 (38.41%)	84,907 (36.41%)	27,821 (43.34%)	16,899 (32.19%)	133 (113)	86 (79)	37 (30)	8 (4)	2
*Paraquilegia microphylla* (Royle) J. R. Drumm. & Hutch.	NC_041479	164,383 (38.87%)	84,925 (36.62%)	30,979 (43.77%)	17,500 (32.42%)	134 (114)	86 (79)	37 (30)	8 (4)	3

All the 11 plastomes each identically encoded 131 predicted functional genes and three pseudo genes, of which seven protein-coding genes, seven tRNA genes, four rRNA genes, and two pseudo genes were duplicated in the IR regions (Figure 1). Two introns were detected in each of two protein-coding genes (*clpP* and *ycf3*) while a single intron was detected in each of 11 protein-coding genes (*atpF*, *ndhA*, *ndhB*, *petB*, *petD*, *rpl2*, *rpl16*, *rpoC1*, *rps12*, *rps16*, and *ycf15*) and six tRNA genes (*trnA^UGC^*, *trnG^UCC^*, *trnI^GAU^*, *trnK^UUU^*, *trnL^UAA^*, and *trnV^UAC^*; Supplementary Table S1). Among 79 protein-coding genes, 75 contained standard AUG as the initiation codon, while three genes (*ndhD*, *rps19*, and *ycf15*) contained GUG instead, and the *rpl2* started with ACG.

### Polymorphic Variation and Hypervariable Regions

**Figure 3:**
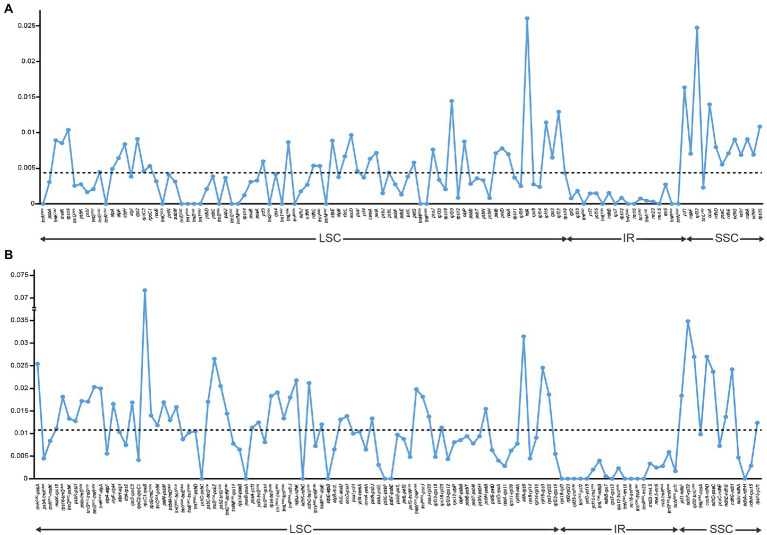
Comparison of nucleotide variability (*π*) values in *Thalictrum* plastomes. **(A)** Pi values among genes, **(B)** Pi values among intergenic spacer (IGS) regions. The break in the middle of the bars indicated that other regions and genes are omitted here. The dot line denoted the average value.

Nucleotide variations among the complete plastid genomes of the 11 *Thalictrum* species were identified to elucidate the level of sequence divergence (Figure 3). The aligned matrix of the 11 *Thalictrum* plastomes (159,334 bp) contained 2,957 single-nucleotide polymorphisms (SNPs) and 1,016 insertion-deletions (indels). The vast majority of SNPs from coding genes were functionally silent (synonymous), while 594 SNPs (43.8%) and six SNPs (0.4%), from altogether 79 coding genes, were missense and nonsense variations (Supplementary Table S2). A total of 549 simple sequence repeats (SSRs) were identified in the 11 *Thalictrum* plastomes with a range of 39 (*Thalictrum petaloideum*) to 60 (*Thalictrum baicalense*) SSRs were detected in each species (Supplementary Table S3), indicating rich polymorphism of the SSRs among plastomes of different species. The SSC regions showed the highest nucleotide diversity (*π* = 0.01381), followed by the LSC (*π* = 0.00803) and IR (*π* = 0.00154) regions. In the 114 unique genes, the nucleotide diversity for each locus ranged from 0 (e.g., *rps7*, *rrn16*, and *trnC^GCA^*) to 0.02608 (*infA*) with an average of 0.00438, whereby 10 regions (i.e., *infA*, *rpl32*, *ycf1*, *rpl20*, *ccsA*, *rpl22*, *rpl16*, *rps15*, *rps16*, and *accD*) had remarkably high values (*π* > 0.0096; Supplementary Table S1; Figure 3A). For exons in genes, the nucleotide diversity ranged from 0 (e.g., *rps7*, *rrn16*, and *trnA-UGC*) to 0.02608 (*infA*) with an average of 0.00373, while for the 115 IGS regions it ranged from 0 (e.g., *atpE-atpB*, *rpl23-trnI^CAU^*, *rrn16-trnI^GAU^*, and *trnI^GAU^-trnA^UGC^*) to 0.03486 (*rpoC1-rpoB*) with an average of 0.01025, except for the *rpoC1-rpoB* (*π* > 0.07171) with a targetable sequence of only 5 bp. Additionally, 10 of those regions showed considerably high values (*π* > 0.0217; i.e., *ndhF-rpl32*, *infA-rps8*, *ccsA-ndhD*, *rpl32-trnL^UAG^*, *trnS^UGA^-psbZ*, *trnH^GUG^-psbA*, *rpl16-rps3*, *ndhG-ndhI*, *ndhD-psaC*, and *ndhJ-ndhK*; see Supplementary Table S4; Figure 3B).

### Phylogenetic Analysis

**Figure 4:**
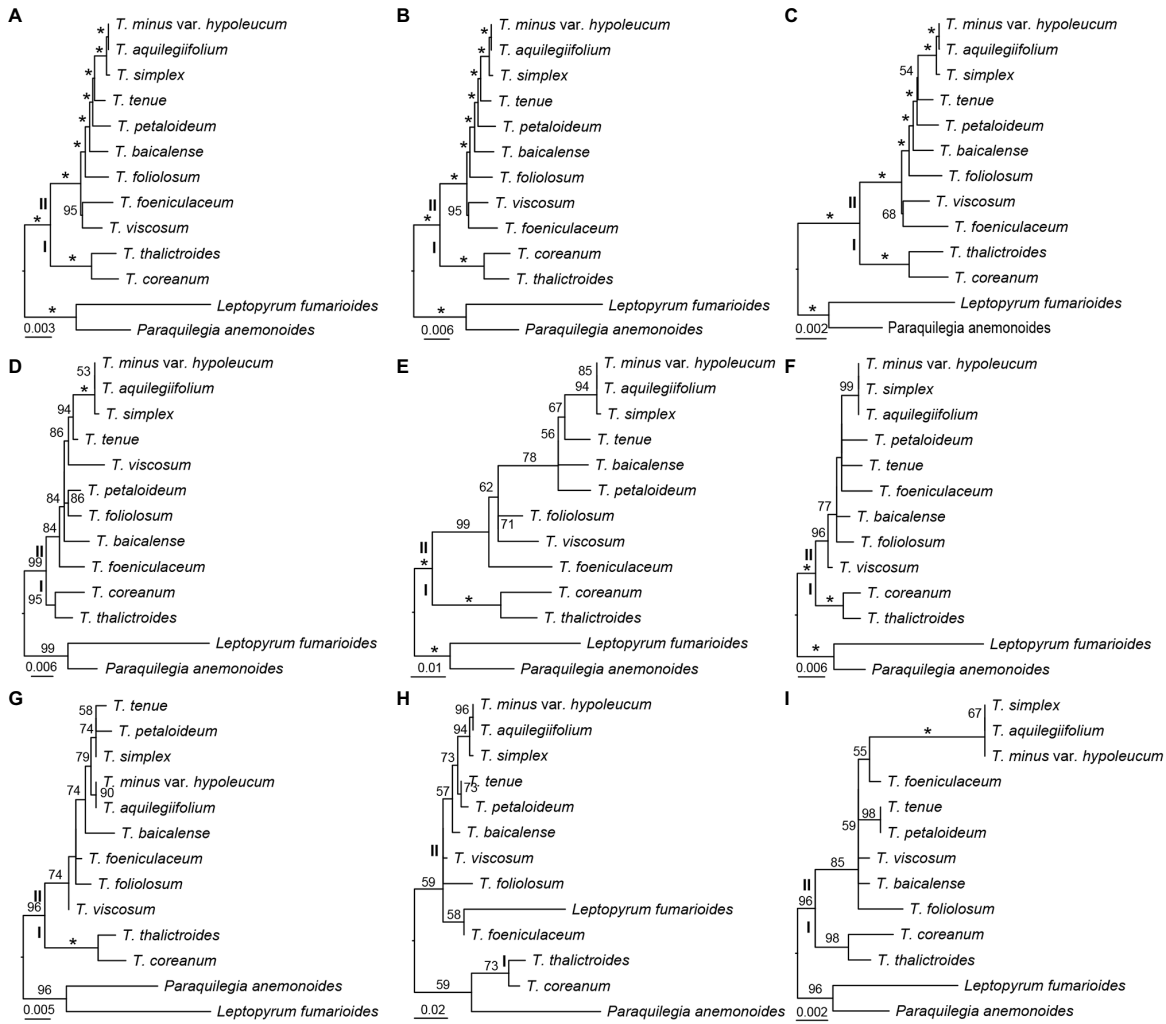
Phylogenetic relationships of *Thalictrum* inferred from maximum likelihood (ML) analysis. **(A)** All sequence, **(B)** concatenation of 115 IGS regions, **(C)** concatenation of 114 gene sequences, **(D)** *rpl16* (with intron, Soza et al., 2012), **(E)** *ndhC-trnV^UAC^* (Soza et al., 2013), **(F)** *ndhA* intron (Wang et al., 2019), **(G)** *trnL^UAA^-trnF^GAA^* (Wang et al., 2019), **(H)** *rpl32-trnL^UAG^* (Wang et al., 2019), and **(I)** *rbcL* (Wang et al., 2019). The numbers above the branches indicate bootstrap support (%), and the asterisk indicates 100% bootstrap support in ML tree.

Three datasets, the whole complete plastid genome sequences, IGS regions, and gene sequences were constructed to investigate the phylogenetic relationships among the 11 *Thalictrum* species, with *P. anemonoides* and *Leptopyrum fumarioides* as two outgroups. By using ML method, three phylogenetic trees were built based on the three respective datasets, whose topologies were found to be highly concordant between one another (Figures 4A–C). The *Thalictrum* was strongly supported as a monophyletic group [bootstrap support (bs) = 100%], and contained two major clades that are strongly supported as sister groups: clades I (bs = 100%) and II (bs = 100%; Figures 4A–C). The resolution of previously used six molecular fragments was also evaluated for *Thalictrum* species. Five genes and/or their introns and spacers yielded similar results except for the *rpl32-trnL^UAG^* (Figures 4D–I). However, different supporting values were observed from the nodes based on different sequence dataset. For example, two nodes in clades II derived from the dataset of gene sequences both showed weaker supports (bs = 54% and bs = 68%; Figure 4C) than those derived from complete plastid genome sequences (bs = 100% and bs = 95%; Figure 4A) and IGS regions (bs = 100% and bs = 95%; Figure 4B). Additionally, the *rpl16* intron had the strongest support within clades II (Figure 4D), while *rbcL* had the weakest support in them (Figure 4I). These results indicated a much stronger resolving power of complete plastid genome sequences as well as IGS and intron regions as compared to the exon regions, which may serve as a reliable source of phylogenetic information in *Thalictrum*.

### Selective Pressure and Codon Usage Analysis

Selective pressure analysis was conducted for CDS of all the 79 plastid protein-coding genes. A total of 66 genes are fit of m1 model in which *atpF* showed the highest *ω* ratio (1.13) except for *rpl23* (*ω* = 999), while other 13 genes (*psbL*, *psaC*, *rps12*, *rps19*, *petB*, *psbN*, *psbF*, *psaJ*, *psbE*, *rpl36*, *psbZ*, *petN*, and *rps7* are fit of m0 model; Table 2). Among the 66 genes, most (50/66) were located in LSC region following by IR (7/66) and SSC (9/66) regions. The values of *ω* are significantly different (*p* < 0.05) between *Thalictrum* species for *ndhG* (SSC), *petA* (LSC), and *rpl22* (LSC) gene based on likelihood ratio tests, within some species have positive selection (e.g., *ndhG* in *T. coreanum*, *T. foeniculaceum*, *Thalictrum foliolosum*, and *T. thalictroides*; *petA* in *T. minus* var. *hypoleucum*). No genes in IR regions were detected significantly different between different species. However, 12 genes (LSC: *atpF*, *rpl33*, *rpl20*, *rps16*, *rps18*, *petG*, *rpl2*, *petL*, *psbJ*, *psbM*; IR: *rpl23*; SSC: *rps15*) were subject to positive selection in most species (median of *ω* > 1; see Table 2; Supplementary Tables S6, S7), although their values of *ω* are not significantly different between different species.

**Table 2:** Summary of models H0 and HA analyzed in the study. *d_N_*, *d_S_*, and *ω* are presented as medians.

Gene	H0: m0				HA: m1				2*(HA-H0)	*p*-value
	*d_N_*	*d_S_*	*ω0*	lnL	*d_N_*	*d_S_*	*ω*	lnL		
*accD*	0.0005	0.0019	0.2489	−2437.30	0.000832	0.00428	0.193579	−2433.08	8.44	0.75
*atpA*	0.0001	0.0021	0.0705	−2272.75	0	0.002509	0.0001	−2266.30	12.90	0.38
*atpB*	0.0002	0.0022	0.0699	−2221.92	0	0.002701	0.0001	−2213.35	17.14	0.14
*atpE*	0	0	0.1804	−642.17	0.000001	0.000005	0.0372454	−635.33	13.69	0.32
*atpF*	0	0	1.1265	−866.78	0.000002	0.000002	124.242	−862.26	9.02	0.70
atpH	0	0	0.0001	−366.55	0.000002	0.000005	0.0001	−366.55	0.000258	1.00
*atpI*	0.0004	0.0043	0.0857	−1137.90	0.000001	0.004595	0.0001	−1130.12	15.55	0.21
*ccsA*	0.0022	0.0071	0.3153	−1836.83	0.002604	0.010913	0.169018	−1831.85	9.97	0.62
*cemA*	0.001	0.0034	0.2884	−1096.08	0.000001	0.000006	0.0001	−1086.23	19.71	0.07
*clpP*	0	0	0.0694	−935.05	0.000001	0.000004	0.0001	−929.55	11.01	0.53
*infA*	0.0096	0.0123	0.7783	−269.79	0.013353	0.000013	74.8743	−264.81	9.97	0.62
*matK*	0.0027	0.0058	0.4631	−2572.82	0.002523	0.006385	0.911524	−2562.74	20.15	0.06
*ndhA*	0.0008	0.0053	0.1467	−1710.61	0.000002	0.003808	0.329949	−1704.63	11.97	0.45
*ndhB*	0	0	0.1178	−2133.67	0	0.000006	0.0001	−2129.17	8.99	0.70
*ndhC*	0	0	0.0916	−566.08	0	0.000005	0.0001	−562.34	7.48	0.82
*ndhD*	0.0011	0.0108	0.0998	−2601.20	0.00089	0.011321	0.0627638	−2593.10	16.20	0.18
*ndhE*	0	0	0.0235	−456.81	0	0.000006	0.0001	−454.86	3.92	0.98
*ndhF*	0.0009	0.0071	0.1292	−2463.23	0.001765	0.007192	0.147131	−2455.95	14.55	0.27
*ndhG*	0.001	0.005	0.1954	−909.26	0.000003	0.000074	0.0001	−896.00	26.52	0.01
*ndhH*	0.0005	0.006	0.0866	−1923.38	0.001108	0.003891	0.0698539	−1919.64	7.48	0.82
*ndhI*	0.0007	0.0061	0.113	−908.57	0.000001	0.008613	0.0001	−902.89	11.35	0.50
*ndhJ*	0	0	0.3505	−688.53	0	0.000005	0.0001	−686.35	4.38	0.98
*ndhK*	0	0	0.3155	−1048.40	0	0.000006	0.0001	−1041.06	14.69	0.26
*petA*	0.0004	0.003	0.1455	−1568.16	0.000002	0.004392	0.0001	−1555.79	24.74	0.02
*petD*	0	0	0.0307	−741.85	0.000001	0.000005	0.0102435	−739.71	4.29	0.98
*petG*	0	0	0.8509	−156.93	0.000002	0	999	−155.55	2.77	1.00
*petL*	0	0	0.1008	−136.46	0.000002	0	313.224	−135.59	1.74	1.00
*psaA*	0	0.0018	0.0084	−3309.32	0	0.001795	0.0001	−3306.01	6.62	0.88
*psaB*	0.0002	0.0031	0.0672	−3293.87	0	0.003743	0.0001	−3286.29	15.16	0.23
*psaI*	0	0	0.8811	−177.10	0	0.000005	0.106004	−174.86	4.48	0.97
*psbA*	0.0001	0.0037	0.0157	−1554.95	0	0.003857	0.0001	−1552.80	4.31	0.98
*psbB*	0.0002	0.0021	0.0878	−2235.59	0	0.002695	0.0001	−2229.89	11.39	0.50
*psbC*	0.0001	0.0024	0.0259	−2138.08	0	0.002509	0.0001	−2131.91	12.33	0.42
*psbD*	0	0	0.075	−1533.73	0	0.000005	0.0001	−1529.39	8.68	0.73
*psbH*	0	0	0.12	−321.61	0.000002	0.000006	0.0978274	−319.64	3.93	0.98
*psbI*	0	0	0.0001	−152.19	0	0.000003	0.0001	−152.19	0.005686	1
*psbJ*	0	0	0.1454	−163.03	0.000002	0	113.399	−162.51	1.05	1.00
*psbK*	0	0	0.3253	−268.48	0	0.000005	0.0001	−265.68	5.59	0.94
*psbM*	0	0	0.0001	−126.66	0.000002	0	212.117	−126.66	0.000884	1.00
*psbT*	0	0	0.245	−142.20	0	0.000004	0.0001	−141.67	1.05	1.00
*rbcL*	0.0004	0.0018	0.1906	−2281.62	0	0.002963	0.0001	−2274.83	13.59	0.33
*rpl14*	0	0	0.037	−529.92	0.000001	0.000006	0.0001	−526.77	6.30	0.90
*rpl16*	0	0	0.1546	−631.29	0.000001	0.000005	0.0001	−624.98	12.62	0.40
*rpl2*	0	0	0.183	−1139.62	0.000002	0	31.3706	−1138.57	2.10	1.00
*rpl20*	0	0	0.4022	−628.11	0.000002	0.000004	9.55435	−623.99	8.24	0.77
*rpl22*	0	0	0.4957	−931.12	0	0.000007	0.0001	−919.97	22.31	0.03
*rpl23*	0	0	999	−396.07	0.000002	0.000001	55.6975	−396.07	0.00	1.00
*rpl33*	0	0	0.1364	−307.30	0.000002	0	491.843	−303.04	8.51	0.74
*rpoA*	0.001	0.006	0.1692	−1751.50	0.000001	0.010121	0.0001	−1742.58	17.85	0.12
*rpoB*	0.0003	0.0029	0.1019	−4816.32	0.000418	0.001825	0.0461232	−4807.91	16.82	0.16
*rpoC1*	0.0007	0.004	0.1796	−3224.37	0.000638	0.002151	0.0743791	−3216.52	15.71	0.20
*rpoC2*	0.0012	0.0042	0.3139	−6585.10	0.001256	0.004513	0.215304	−6576.06	18.07	0.11
*rps11*	0	0	0.0797	−634.65	0	0.000005	0.0001	−631.31	6.68	0.88
*rps14*	0	0	0.1102	−420.73	0	0.000005	0.0001	−418.47	4.51	0.97
*rps15*	0	0	0.6425	−434.79	0.000002	0	76.3582	−430.76	8.08	0.78
*rps16*	0	0	0.4244	−380.25	0.000002	0	15.7794	−377.71	5.07	0.96
*rps18*	0	0	0.0918	−411.02	0.000002	0	192.024	−408.76	4.52	0.97
*rps2*	0	0	0.4331	−1203.16	0.000002	0.000006	0.134231	−1198.26	9.80	0.63
*rps3*	0.0005	0.005	0.1081	−1071.79	0.000001	0.005949	0.0001	−1064.71	14.16	0.29
*rps4*	0.0007	0.0051	0.1334	−944.70	0.000001	0.000006	0.0001	−937.89	13.61	0.33
*rps8*	0	0	0.2502	−588.72	0	0.000006	0.0001	−586.69	4.08	0.98
*ycf1*	0.0035	0.0068	0.5214	−11057.96	0.003664	0.004828	0.495128	−11050.66	14.59	0.26
*ycf2*	0	0	0.3282	−246.36	0	0.000005	0.0001	−244.96	2.79	1.00
*ycf3*	0	0	0.1983	−696.68	0.000001	0.000004	0.20605	−693.90	5.54	0.94
*ycf4*	0	0	0.036	−802.98	0	0.000005	0.0001	−799.72	6.51	0.89
*ycf15*	0.0001	0.0002	0.552	−9655.78	0.000185	0.000006	0.402698	−9648.33	14.90306	0.246781
*petB*	0	0.0058	0.0001	−991.11	0.000001	0.005832	0.0001	−991.11	0.000242	1
*petN*	0	0	0.2706	−116.86	0.000002	0	198.276	−116.86	0.001416	1
*psaC*	0	0	0.0001	−375.50	0	0.000005	0.0001	−375.50	0.00008	1
*psaJ*	0	0	0.0001	−208.38	0	0.000004	0.0001	−208.38	0.00036	1
*psbE*	0	0	0.0001	−350.96	0	0.000005	0.0001	−350.96	0.000432	1
*psbF*	0	0	0.0001	−159.00	0	0.000005	0.0001	−159.00	0.000294	1
*psbL*	0	0	0.0001	−162.62	0	0.000007	0.0001	−162.62	0	1
*psbN*	0	0	0.0001	−180.08	0	0.000004	0.0001	−180.08	0.000258	1
*psbZ*	0	0	0.0001	−247.62	0	0.000005	0.0001	−247.62	0.000616	1
*rpl36*	0	0	0.0001	−152.86	0.000001	0.000002	0.520692	−152.86	0.00048	1
*rps12*	0	0	0.0001	−496.22	0.000002	0	60.2915	−496.22	0.000092	1
*rps19*	0	0	0.0001	−404.64	0.000001	0.000005	0.0001	−404.64	0.000202	1
*rps7*	0	0	0.0001	−612.36	0.000002	0	78.7221	−612.36	0.002164	1

**Figure 5:**
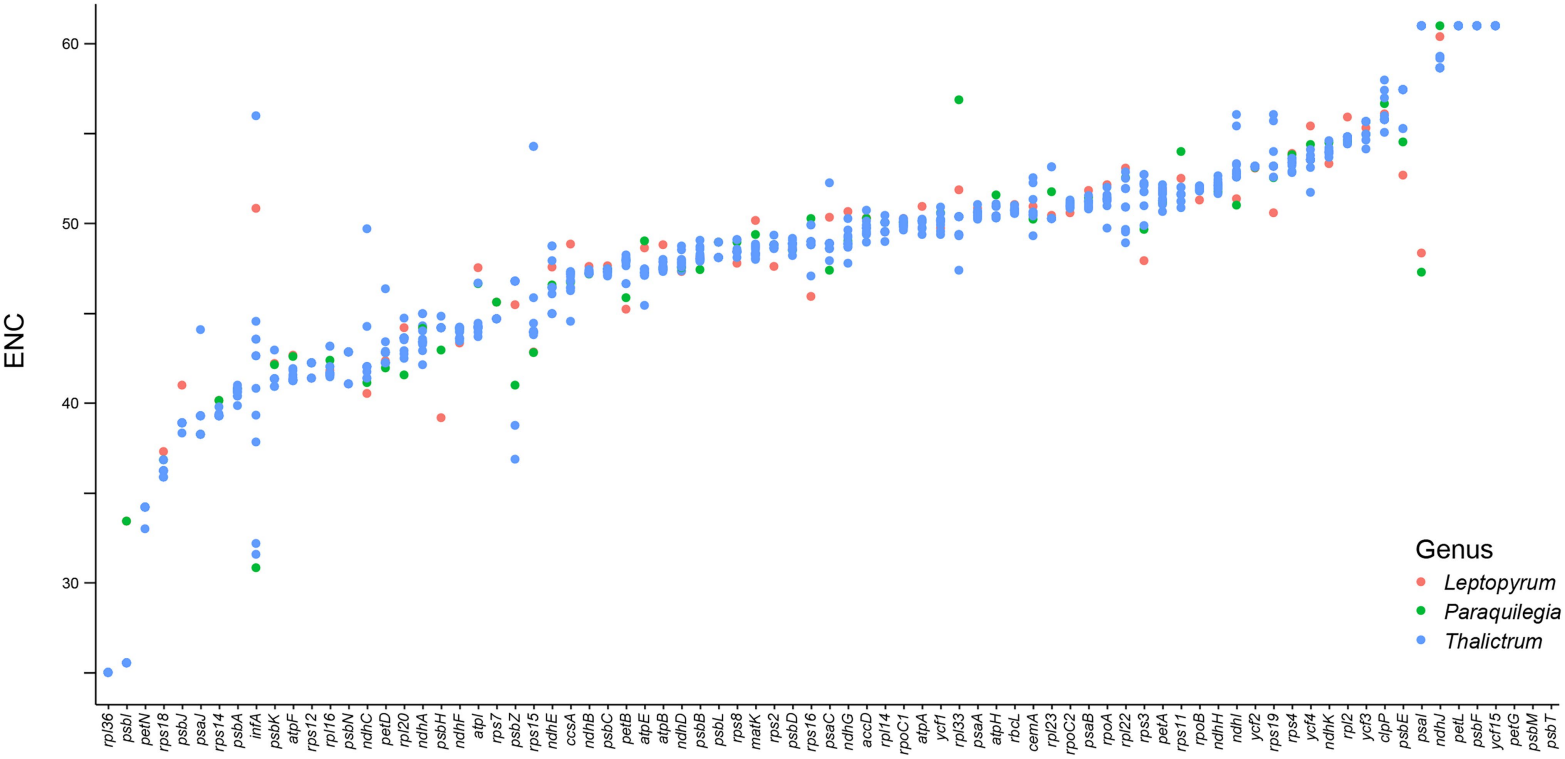
The effective number of codons in the study.

We further analyzed the codon usage bias of the 79 protein coding genes in the plastomes of the 11 *Thalictrum* species. Most codons (55/64) were found to be used without bias or with only a slight bias (0.5 ≤ RSCU ≤ 1.5) in the protein-coding genes (Supplementary Table S8). The effective number of codons (ENC) and codon bias index (CBI) of all the 79 genes varied within a wide range, e.g., from 25.02 to 61.00 and from 0.28 to 0.85, respectively, with a median value of 49.0 and 0.50, respectively (Figure 5; Supplementary Figure S1; Supplementary Table S8). The data indicated that these genes were probably expressed in different levels due to their different usage frequencies of the rare and optimal codons, although they are all highly conserved in the plastomes. Most genes in SSC region (80.0%) showed relatively strong bias in the codon usage (ENC ≤ ENC*_median_* = 49.0 or CBI ≥ CBI*_median_* = 0.5), while 67.2% of genes in LSC region and 50.0% of genes in IR region performed relatively strong codon usage bias. Notably, almost all genes under positively selective pressures in more than half species performed relatively strong bias in the codon usage (ENC ≤ ENC*_median_* = 49.0 or CBI ≥ CBI*_median_* = 0.5), e.g., *atpF* featured a relatively strong codon usage bias with a low ENC of 41.61. This finding suggested that those important genes with higher expression levels may played important roles in the evolution and divergence of *Thalictrum* plastomes.

## Discussion

### Plastome Characteristics of *Thalictrum*

In the present study, complete plastome sequences were firstly assembled for *T. minus* var. *hypoleucum* and *T. simplex* in the *Thalictrum* genus, with a total length of 156,211 and 156,258 bp, respectively (Table 1). The two plastomes are also highly similar in overall structure and gene order when compared to the majority of previously published plastomes of other nine species in *Thalictrum* (Park et al., 2015; He et al., 2019, 2021b; Morales-Briones et al., 2019). However, there was obvious variation in the IR-SC boundary regions among the 11 *Thalictrum* plastomes (Figure 2). The variations in IR-SC boundary regions in the 11 *Thalictrum* plastomes led to their length variation of the four regions and whole genome sequences. The expansion and contraction of the IR-SC boundary regions was considered as a primarily mechanism causing the length variation of angiosperm plastomes (Kim and Lee, 2004). In general, such expansions or contractions of the IRs into or out of adjacent single-copy regions are frequently observed in angiosperm plastomes (e.g., Yang et al., 2016; Zhang et al., 2016; Ye et al., 2018).

Nonetheless, there are particular genes, especially *ycf1*, *rps19*, *ndhF*, *ycf15*, and ψ*rpl32*, which deserve closer scrutiny. For instance, in various members of *Thalictrum*, *ycf1* is duplicated, with a shorter copy (ψ*ycf1*, 1,144–1,152 bp) and a larger copy (*ycf1*, 5,616–5,658 bp) located at the SSC-IR_A_ and SSC-IR_B_ boundaries, respectively (Figure 2). Similarly, the *rps19* is present as two copies including ψ*rps19* (100–122 bp) and *rps19* (279 bp) at the SSC-IR_B_ and SSC-IR_A_ boundaries respectively except in *T. thalictroides* (Figure 2). Both shorter copies apparently resulted from incomplete duplication. Similar pseudogenizations of *ycf1* and locations of ψ*ycf1* copies are known from other plants (Yang et al., 2013, Szczecińska and Sawicki, 2015; Ye et al., 2018), and two copies of *rps19* have been found in Podophylloideae (Berberidaceae; Ye et al., 2018). As for the *ndhF*, the coding sequence was unexpectedly terminated by a stop-codon-gained event caused by nucleotide variation of a poly-A region in eight *Thalictrum* species except for *T. coreanum*, *T. foeniculaceum*, and *T. thalictroides*. For the *ycf15*, an intact copy and an interrupted gene have been found in other plants, with lengths of c. 150–300 bp (Raubeson et al., 2007, Shi et al., 2013). By contrast, an interrupted *ycf15* gene has been annotated in the sequenced chloroplast genomes in *Thalictrum* species. Additionally, ψ*rpl32* is incomplete because the *rpl32* gene was found to be transferred to the nucleus in the ancestor of the subfamily Thalictroideae (Park et al., 2015).

Regarding the initiation codon, *ndhD*, *rps19*, and *ycf15* used GUG, while *rpl2* used ACG in *Thalictrum*. The ACG codon may be restored to the canonical start codon (AUG) by RNA editing (Hoch et al., 1991; Takenaka et al., 2013), whereas GUG has been detected in in other plastomes (Kuroda et al., 2007; Gao et al., 2009; Zhang et al., 2016).

### Noncoding Regions as a Source of Phylogenetic Information in *Thalictrum*

Given that the nuclear-genome coded ITS region is often presented as more than one copy in *Thalictrum*, sequences of cpDNA intergenic spacers have been employed to uncover intraspecific variability in *Thalictrum* (Soza et al., 2012, 2013). The IRs usually showed lower sequence divergence than the SC regions in most of higher plants and possibly due to copy correction between IR sequences by gene conversion (Khakhlova and Bock, 2006; Zhang et al., 2016). In the present study, the whole genome and IGS regions manifested higher sequence divergence than genes did, and genes with introns showed higher sequence divergence than genes without introns in *Thalictrum* species (Figure 3). In general, the non-coding regions (introns and spacers) had higher variability proportions than coding regions, which was also true for most higher plants (Shaw et al., 2014; Zhang et al., 2016).

In some studies, eight noncoding regions (*ndhF-rpl32*, *rpl32-trnL^UAG^*, *ndhC-trnV-UAC*, *rps16-trnQ^UUG^*, *psbE-petL*, *trnT^GGU^-psbD*, *petA-psbJ*, and *rpl16* intron) have been identified as the best possible choices for low-level phylogenetic studies on angiosperms (Shaw et al., 2014). Among these regions, *ndhF-rpl32*, *rpl32-trnL^UAG^*, and the *rpl16* intron were also identified as highly divergent loci among *Thalictrum* species in the present study. Nonetheless, two IGS regions related to *rpl32* are not suitable as molecular markers in *Thalictrum* because the *rpl32* gene is often transferred to the nucleus (Park et al., 2015). Aside from these loci, we also observed high nucleotide diversity in *infA-rps8*, *ccsA-ndhD*, *trnS^UGA^-psbZ*, *trnH^GUG^-psbA*, *rpl16-rps3*, *ndhG-ndhI*, and *ndhD-psaC* regions. Additionally, an intron of *rps16* also showed highly variable here, similarly to Podophylloideae (Berberidaceae; Ye et al., 2018). These divergence hotspot regions of the 11 *Thalictrum* plastid genome sequences provided abundant information for developing effective molecular markers to the phylogenetic analyses and plant identification of *Thalictrum* species. Besides, the resolution and efficiency of chloroplast markers can be strongly affected by the length of target fragment. The *rpoC1-rpoB* region has a relatively high nucleotide diversity among different plastomes, but cannot be a good molecular marker as its target length is only 5 bp.

### Phylogenetic Relationships

The plastid genome sequences have been utilized successfully for the phylogenetic studies on angiosperms (Jansen et al., 2007; Huang et al., 2014; Kim et al., 2015; Li et al., 2019). Our phylogenetic trees based on whole complete plastid genome sequences, 116 IGS regions, and 114 gene sequences revealed that *Thalictrum* contains two major clades that is consistent with previous studies (Figures 4A–C; Soza et al., 2012, 2013; Morales-Briones et al., 2019; Wang et al., 2019). However, the relationships along the backbone of the clades are not well-supported in their studies. None of the sections traditionally circumscribed for this genus (Tamura, 1995) is monophyletic. It is necessary to apply more samplings and find more efficient molecular markers for *Thalictrum*.

Our phylogenetic trees indicated that 116 IGS regions had stronger support than 114 gene sequences (Figures 4B,C). Additionally, the *rpl16* intron—that was used by Soza et al. (2012) with high sequence divergence in the studies—showed also strong support in clades II here (Figure 4D). While the coding regions of *rbcL* employed by Wang et al. (2019) showed lower supports within clades II in our analysis (Figure 4I). The non-coding regions (introns and spacers) are more variable molecular markers. For the ML tree of *rpl32-trnL^UAG^* used by Wang et al. (2019), the outgroups are embedded in *Thalictrum* probably because the matrix of *rpl32-trnL^UAG^* contained lots of indels (Figure 4H). The *rpl32* gene is often transfers to the nucleus (Park et al., 2015) that make the *ndhF-rpl32*, *rpl32-trnL^UAG^*, and *rpl32* regions not reliable to be markers for phylogeny in *Thalictrum*.

### Positive Selection in Different Genes

It is believed that selection is the most probable components of the evolutionary forces acting on most highly expressed genes, although all genes are basically subjected to a certain degree of natural selection (Gouy and Gautier, 1982; Sueoka, 1999; Sharp et al., 2010). And the degeneracy of genetic code leads to the expression of variation contained in a gene through its manifestation in protein, which varied among different species (Edelman and Gally, 2001; Wan et al., 2004; Chakraborty et al., 2020). In the present study, we observed different codon usage frequency on different genes under positive pressure. For example, 12 plastid genes (*atpF*, *rpl33*, *rps15*, *rpl20*, *rps16*, *rps18*, *petG*, *rpl2*, *petL*, *psbJ*, *psbM*, and *rpl23*) were observed under positive selective pressure in most of the 11 *Thalictrum* species among which 11 showed relatively higher CBI values (>0.5) suggesting high expression level *in vivo*; while three plastid genes that are relative with NADH oxidoreductase (*ndhG*), cytochrome b6/f complex (*petA*), and ribosomal proteins (*rpl22*) were observed under significantly strong positive selective pressure (*p* < 0.05 based on likelihood ratio tests) in only 1–4 *Thalictrum* species, showing relatively lower CBI (<0.5). The former and latter genes performed different codon usage bias suggesting different expression levels due to different usage frequency of the rare and optimal codons, which could further affected the functional patterns of those genes during their evolution process. Additionally, it also indicated potential functional divergence among plastid genomes of different *Thalictrum* species, according to abundant differences observed between selective pressures and usage codon frequencies for different plastid genes in these species.

## Conclusion

This is the first report to describe a comprehensive landscape of plastomic variations among *Thalictrum* species on the basis of 11 complete plastomes. Comparison between these plastomes uncovered not only high similarities in overall structure, gene order, and content but also some structural variations caused by the expansion or contraction of the IR regions into or out of adjacent single-copy regions. DNA sequence divergence across 11 *Thalictrum* plastomes revealed that *infA-rps8*, *ccsA-ndhD*, *trnS^UGA^-psbZ*, *trnH^GUG^-psbA*, *rpl16-rps3*, *ndhG-ndhI*, *ndhD-psaC*, and *ndhJ-ndhK* are among the fastest-evolving loci and are promising molecular markers. Therefore, these highly variable loci should be valuable for future phylogenetic and phylogeographic studies on *Thalictrum*. Our phylogenomic analyses based on whole complete plastid genome sequences, 116 IGS regions and 114 gene sequences were all supported the monophyly of *Thalictrum* and two major clades within this genus. Furthermore, among 79 plastome-derived protein-coding genes (CDSs), 15 genes were identified as fast evolving genes, which were all proved to be under positive selection but showed different bias in their codon usage frequencies. Overall, our results demonstrate the ability of plastid phylogenomics to improve phylogenetic resolution, and will expand the understanding of plastid gene evolution in *Thalictrum*.

## Data Availability Statement

All raw sequencing reads generated in the study have been deposited in NCBI under the BioProject accession PRJNA817687. The complete sequences and annotations of plastomes have also been deposited at GenBank under the accessions OM501079 and OM501080. The updated annotations of plastomes for the other 11 species in this study have been deposited to the Figshare online database (https://doi.org/10.6084/m9.figshare.19108097.v1).

## Author Contributions

W-CH and Z-QW conceived the research. H-WP carried out taxon sampling and generated all the data. K-LX and W-CH performed the data analyses. K-LX, W-CH, and WM wrote the manuscript with help from Z-QW. Y-XY revised the manuscript. All authors contributed to the article and approved the submitted version.

## Funding

This work was supported by the China Postdoctoral Science Foundation (grant number 2021M703540), the Training of Excellent Science and Technology Innovation talents in Shenzhen-Basic Research on Outstanding Youth (grant number RCYX20200714114538196), the National Natural Science Foundation of China (grant number 32011530072), the Initial fund of Shenzhen Agricultural Genome Research Institute, Chinese Academy of Agricultural Sciences (grant number SJXW19073), and the Russian Science Foundation [grant number 19-74-10082 (preparation of material)], within state assignments for CSBG SB RAS [grant number АААА-А21-121011290024-5 (study of herbarium collections)].

## Conflict of Interest

The authors declare that the research was conducted in the absence of any commercial or financial relationships that could be construed as a potential conflict of interest.

## Publisher’s Note

All claims expressed in this article are solely those of the authors and do not necessarily represent those of their affiliated organizations, or those of the publisher, the editors and the reviewers. Any product that may be evaluated in this article, or claim that may be made by its manufacturer, is not guaranteed or endorsed by the publisher.

## Footnotes

^1^ https://irscope.shinyapps.io/irapp/
