# Corrigendum: Organization, phylogenetic marker exploitation, and gene evolution in the plastome of *Thalictrum* (Ranunculaceae)

**DOI:** 10.3389/fpls.2023.1303516

**Published:** 2023-10-18

**Authors:** Kun-Li Xiang, Wei Mao, Huan-Wen Peng, Andrey S. Erst, Ying-Xue Yang, Wen-Chuang He, Zhi-Qiang Wu

**Affiliations:** ^1^ Shenzhen Branch, Guangdong Laboratory of Lingnan Modern Agriculture, Genome Analysis Laboratory of the Ministry of Agriculture and Rural Affairs, Agricultural Genomics Institute at Shenzhen, Chinese Academy of Agricultural Sciences, Shenzhen, China; ^2^ State Key Laboratory of Systematic and Evolutionary Botany, Institute of Botany, Chinese Academy of Sciences, Beijing, China; ^3^ College of Ecology and Environment, Hainan University, Haikou, China; ^4^ College of Life Sciences, University of Chinese Academy of Sciences, Beijing, China; ^5^ Central Siberian Botanical Garden, Russian Academy of Sciences, Novosibirsk, Russia; ^6^ Laboratory Herbarium (TK), Tomsk State University, Tomsk, Russia; ^7^ Kunpeng Institute of Modern Agriculture at Foshan, Chinese Academy of Agricultural Sciences, Foshan, China

**Keywords:** *Thalictrum*, plastid genome, genome structure, molecular markers, phylogeny

**Error in Author List**

In the published article, there was an error in the **author list**. Authors Ying-Xue Yang, Wen-Chuang He and Zhi-Qiang Wu did not have an indication of equal contribution.

The corrected author list appears below:

Kun-Li Xiang^1,2^, Wei Mao^3^, Huan-Wen Peng^2,4^, Andrey S. Erst^5,6^, Ying-Xue Yang^1 *†^, Wen-Chuang He^1 *†^ and Zhi-Qiang Wu^1,7 *†^

^1^Shenzhen Branch, Guangdong Laboratory of Lingnan Modern Agriculture, Genome Analysis Laboratory of the Ministry of Agriculture and Rural Affairs, Agricultural Genomics Institute at Shenzhen, Chinese Academy of Agricultural Sciences, Shenzhen, China, ^2^State Key Laboratory of Systematic and Evolutionary Botany, Institute of Botany, Chinese Academy of Sciences, Beijing, China, ^3^College of Ecology and Environment, Hainan University, Haikou, China, ^4^College of Life Sciences, University of Chinese Academy of Sciences, Beijing, China, ^5^Central Siberian Botanical Garden, Russian Academy of Sciences, Novosibirsk, Russia, ^6^Laboratory Herbarium (TK), Tomsk State University, Tomsk, Russia, ^7^Kunpeng Institute of Modern Agriculture at Foshan, Chinese Academy of Agricultural Sciences, Foshan, China

*Correspondence: Zhi-Qiang Wu, wuzhiqiang@caas.cn; Wen-Chuang He, hewenchuang@caas.cn; Ying-Xue Yang, yyxue32@163.com

†These authors have contributed equally to this work and share last authorship.

The authors apologize for this error and state that this does not change the scientific conclusions of the article in any way. The original article has been updated.

