# Supplementary material for: Organization, Phylogenetic Marker Exploitation, and Gene Evolution in the Plastome of Thalictrum (Ranunculaceae)
Source: Front Plant Sci. 2022 May 20;13:897843. doi: 10.3389/fpls.2022.897843 (PMC9166237; doi:10.3389/fpls.2022.897843)
Supplement: Supplementary file 1 [file Data_Sheet_1.ZIP › Supplementary materials/Supplementary materials-Revison.pdf]

**Supplementary Materials**

**Fig. S1.** The coefficient of variation for the the codon usage bias in the study.

**Table S1** Summary of characteristics of 114 genes in chloroplast genomes sequnences in *Thalictrum*.

**Table S2** Functional annotaitons for the SNVs detected in the *Thalictrum* plastomes. The variation types here are defined by snpEff software.

**Table S3** Development of high polymorphism potential SSR markers in *Thalictrum* plastomes.

**Table S4** Summary of characteristics of 115 IGS regions in chloroplast genomes sequnences in *Thalictrum*.

**Table S5** The $d_N$ and $d_S$ under the model 0 (H0) in the study.

**Table S6** The $d_N$, $d_S$ and $\omega$ under the model 1 (HA) in the study.

**Table S7** Relative synonymous codon usage (RSCU) of the AAs used in the *Thalictrum* plastomes.

**Table S8** Summary of the codon usage bias in the study

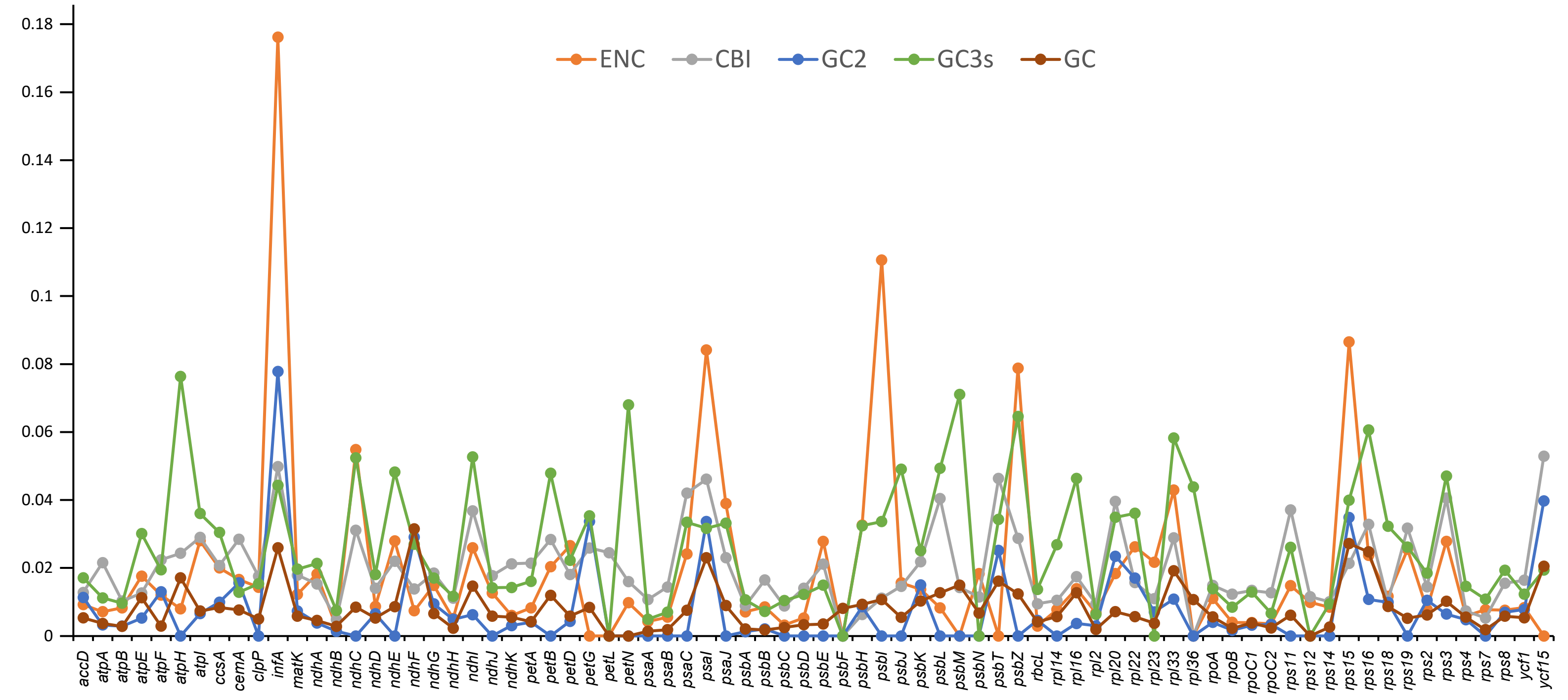
ENC
CBI
GC2
GC3s
GC
0.18
0.16
0.14
0.12
0.1
0.08
0.06
0.04
0.02
0
accD
atpA
atpB
atpE
atpF
atpH
atpI
ccsA
cemA
clpP
infA
matK
ndhA
ndhB
ndhC
ndhD
ndhE
ndhF
ndhG
ndhH
ndhI
ndhJ
ndhK
petA
petB
petD
petG
petL
petN
psaA
psaB
psaC
psaI
psaJ
psbA
psbB
psbC
psbD
psbE
psbF
psbH
psbI
psbJ
psbK
psbL
psbM
psbN
psbT
psbZ
rbcL
rpl14
rpl16
rpl2
rpl20
rpl22
rpl23
rpl33
rpl36
rpoA
rpoB
rpoC1
rpoC2
rps11
rps12
rps14
rps15
rps16
rps18
rps19
rps2
rps3
rps4
rps7
rps8
ycf1
ycf15
